# Accelerated Simplified Swarm Optimization with Exploitation Search Scheme for Data Clustering

**DOI:** 10.1371/journal.pone.0137246

**Published:** 2015-09-08

**Authors:** Wei-Chang Yeh, Chyh-Ming Lai

**Affiliations:** Department of Industrial Engineering and Engineering Management, National Tsing Hua University, Hsinchu City, Taiwan; Jiangnan University, CHINA

## Abstract

Data clustering is commonly employed in many disciplines. The aim of clustering is to partition a set of data into clusters, in which objects within the same cluster are similar and dissimilar to other objects that belong to different clusters. Over the past decade, the evolutionary algorithm has been commonly used to solve clustering problems. This study presents a novel algorithm based on simplified swarm optimization, an emerging population-based stochastic optimization approach with the advantages of simplicity, efficiency, and flexibility. This approach combines variable vibrating search (VVS) and rapid centralized strategy (RCS) in dealing with clustering problem. VVS is an exploitation search scheme that can refine the quality of solutions by searching the extreme points nearby the global best position. RCS is developed to accelerate the convergence rate of the algorithm by using the arithmetic average. To empirically evaluate the performance of the proposed algorithm, experiments are examined using 12 benchmark datasets, and corresponding results are compared with recent works. Results of statistical analysis indicate that the proposed algorithm is competitive in terms of the quality of solutions.

## Introduction

Cluster analysis is principally used to explore useful knowledge, particularly inherent structure, hidden in a dataset with scarce pre-existent information. Such technique has been frequently applied in many complicated tasks, including pattern recognition, image analysis, facility location, and other fields of engineering [1–2]. Cluster analysis aims to categorize unlabeled data into different clusters on the basis of the similarity between data instances. Similarity is generally measured by the distance metric and can also be treated as an optimization problem that requires an optimal assignment of objects to clusters by minimizing the sum of distance metric between each object and its cluster centroid [3].

K-means (KM) clustering, which uses distance metric to partition data into *K* clusters, is common and fundamental because of its simplicity and efficiency. However, the initial state may cause the algorithm to be trapped in local optima, thereby affecting the quality of the solution [4]. Recent studies have made significant progress in overcoming the drawback of KM clustering, particularly by using evolutionary algorithms, including genetic algorithm [5–6], tabu search approach [7], ant colony optimization [8–9], artificial bee colony algorithm [10], and particle swarm optimization [11–13]. Several algorithms inspired by physical phenomenon have also been employed. These algorithms include simulated annealing algorithm [14], big bang-big crunch optimization [15], gravitational search algorithm combined with KM (GSA-KM) [16], and black hole algorithm (BH) [3].

Those approaches may take time to converge in dealing with clustering problems, particularly large problems, because the initial particles are randomly generated and the subsequent updates are probabilistic. To speed up the convergence rate, Krishna and Murty [5] have employed an accelerated strategy called KM operator (KMO), which merges the principles of KM into the clustering algorithm to efficiently find an effective solution. KMO uses arithmetic average to determine new cluster centers in each generation. However, KMO may be disabled after a number of generations. In this case, the computation cost is wasted, and the diversity of population is restricted by the arithmetic average. Some clustering algorithms have combined with the KM by using the result of KM as one of the initial solutions to speed up the convergence rate [9, 13, 16]. However, the outcome of KM heavily depends on the initial choice of the cluster centers and may converge to the local optima rather than global optima. As a result, these hybrid algorithms may start searching from a local optimum and obtain poor-quality solutions.

To overcome such problem, this study proposes an improved simplified swarm optimization (SSO) that combines variable vibrating search (VVS) and rapid centralized strategy (RCS) to solve clustering problems. VVS is an exploitation search scheme which can search nearby for the global best position to refine the quality of the solution by using vibrated movement. It obtains a balance between exploration and exploitation by introducing a function of time (*t*). RCS is modified from KMO and stochastically activated to reduce the computation cost and the loss of the population diversity. To evaluate the proposed algorithm, 10 benchmark datasets are tested, and the performance is compared with state-of-the-art works. Encouraging results are found in terms of efficiency and effectiveness of the proposed algorithm.

The remainder of this paper is organized as follows: Section 2 briefly describes the clustering problem and SSO. Section 3 introduces the proposed algorithm including VVS and RCS. Section 4 presents and discusses the computational results as well as the statistical analysis. Finally, conclusions are summarized in Section 5.

## Related works

### Clustering problem

The goal of clustering is to partition a given set of *N* objects *Y =* {*Y*_1_, *Y*_2_,…, *Y*_*N*_}, each *Y*_*i*_ *=* {*y*_1_, *y*_2_,…, *y*_*D*_} ∈ *R* ^*D*^, into *K* groups, also called clusters *C =* {(*C*_1_, *Z*_1_), (*C*_2_, *Z*_2_),…, (*C*_*K*_, *Z*_*K*_)}, where *C*_*k*_ represents the *k*th cluster, *Z*_*k*_ represents the centroid of the *k*th cluster, and *K* ≤ *N*. The cluster structure is represented as follows [2]:

$$1.\;C_{k}\neq \phi ,k=1,2,\ldots ,K;$$ (1)

$$2.\;{⋃}_{k=1}^{K}C_{k}=Y;$$ (2)

$$3.\;C_{i}\cap C_{j}=\phi ;i,j=1,2,\ldots ,K\;\text{and}\;i\neq j.$$ (3)

To split the objects into different clusters, many similarity criteria have been used in this task. One of most popular criterion is the Euclidean distance metric [3, 7, 9, 11, 16]. KM is an efficient and common clustering method which adopts this metric. The steps of this algorithm are as follows [17]:

Step 1: Randomly choose the *K* initial centroids of clusters, *Z =* {*Z*_1_, *Z*_2_,…, *Z*_*K*_}.
Step 2: Measure the similarity by the Euclidean distance metric, and allocate object *Y*_*i*_ to *C*_*k*_, if *||Y*_*i*_, *Z*_*k*_||^2^ ≤ *||Y*_*i*_, *Z*_*p*_||^2^, *p* = 1, 2,…, *K*, and *k* ≠ *p*.
Step 3: Evaluate the objective function named the sum of intra-cluster distances (SICD), as follows:

$$f(Y,C)=\sum_{k=1}^{K}\sum_{Y_{i}\in C_{k}}{\left\|Y_{i},Z_{k}\right\|}^{2}$$ (4)

Step 4: Recalculate new centroids of clusters, as follows:

$$Z_{new,k}=\frac{1}{N_{k}}\sum_{\forall Y_{i}\in C_{k}}Y_{i}$$ (5)

Step 5: If *Z*_*new*,*k*_ = *Z*_*k*_, then halt. Otherwise, continue from Step 2.

KM regards the clustering problem, which is an NP problem [18], as an optimization problem and aims at minimizing SICD, by assigning objects to the closest cluster centroid. KM is a well-known method in dealing with clustering problems because of its simplicity and efficiency. However, it suffers from the local optimum. This work introduces a novel SSO-based algorithm using the concept of KM to find optimal centroids by minimizing SICD without the initialization problem of KM.

### Simplified swarm optimization

SSO is a population-based algorithm proposed by Yeh in 2009 [19] to compensate for the deficiencies of PSO in solving discrete problems. This algorithm has recently been applied in many research areas because of its simplicity, efficiency, and flexibility [20–22].

In SSO, each individual in the swarm, called a particle representing a solution, is encoded as a finite-length string with a fitness value. Similar to many population-based algorithms, SSO improves the solution of a specified problem by the update mechanism (UM), which is the core of any evolutionary algorithm scheme. The UM of SSO is as follows:

$$x_{ij}^{t}=\left\{ \begin{array}{l} x_{ij}^{t-1}\;if\;\rho \in [0,C_{w})\\ p_{ij}^{t-1}\;if\;\rho \in [C_{w},C_{p})\\ g_{j}\;if\;\rho \in [C_{p},C_{g})\\ x\;if\;\rho \in [C_{g},1] \end{array}\right.$$ (6)

where $x_{ij}^{t}$ is the position value in the *i*th particle with respect to the *j*th variable of the solution space at generation *t*. *p*_*i*_ *=* (*p*_i1_, *p*_i2_,…, *p*_*id*_), where *d* is the total number of variables in the problem domain, represents the best solution with the best fitness value in its own history, known as *pBest*. The best solution with the best fitness value among all solutions is called *gBest*, which is denoted by *g* = (*g*_1_, *g*_2_,…, *g*_*d*_), and *g*_*j*_ denotes the *j*th variable in *gBest*. *x* is a new randomly generated value between the lower bound and the upper bound of the *j*th variable. *ρ* is a uniform random number between 0 and 1. *C*_*w*_, *C*_p,_ and *C*_*g*_ are three predetermined parameters that form four interval probabilities. Thus, *c*_*w*_ *= C*_*w*_, *c*_*p*_ *= C*_*p*_ *-C*_*w*_, *c*_*g*_ *= C*_*g*_ *-C*_*p*_ and *c*_*r*_ *=* 1*-C*_*g*_ represent the probabilities of the new variable from four sources, namely, the current solution, *pBest*, *gBest*, and a random movement in the UM, respectively. The UM updates each particle to be a compromise of those four sources, particularly a random movement, which is different from the original PSO, maintains population diversity, and enhances the capacity of escaping from a local optimum.

## Proposed methods

The proposed algorithm is based on the original SSO, and combines with variable vibrating search (VVS) and rapid centralized strategy (RCS). This section introduces VVS, RCS and overall procedure for the proposed algorithm to solve the clustering problem.

### SSO clustering algorithm

**Fig 1:**
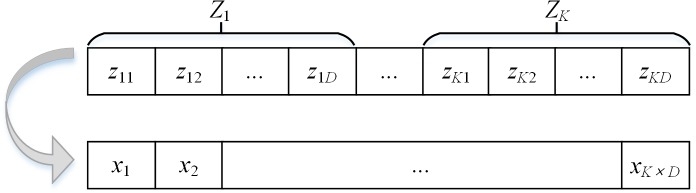
Example of a solution string.

Similar to many population-based algorithms, the SSO clustering algorithm randomly generates a population of particles, also called solutions. Encoding solution is the critical first step toward becoming a clustering algorithm. Assume that a clustering problem with *D* features is partitioned into *K* clusters, then *Z =* {*Z*_1_, *Z*_2_,…, *Z*_*K*_} represents centroid vector and *Z*_*k*_ = {*z*_k1_, *z*_k2_,…, *z*_*kD*_}, where *k* = 1, 2,…, *K*. Therefore, each solution string can be defined as *X* = {*x*_1_, *x*_2_,…, *x*_K×D_} as illustrated in Fig 1.

The primary steps of the SSO for clustering are summarized as follows:

Step 1: Generate a population of particles that represent centroids of each cluster with random positions based on a given dataset.
Step 2: Evaluate the fitness value for each particle in the population according to Eq (4).
Step 3: Update *pBest* and *gBest* if necessary.
Step 4: Update the particle’s variables according to Eq (6).
Step 5: Stop the algorithm if the maximum number of iterations is met; otherwise, return to step 2.

### Variable vibrating search (VVS)

The UM promotes SSO to be an algorithm with the advantages of simplicity and flexibility. However, the UM is a stochastic process with insufficient capability to find nearby extreme points that can affect the efficiency and robustness of SSO, particularly in continuous problems. One way to improve the performance of SSO is adding the local search operation [23]. This modified SSO, called SSO-ELS, uses exchange local search scheme to find a new *pBest* of the particle or a new *gBest* by exchanging attributes with two randomly chosen particles in population. However, it consumes more time than the original SSO.

**Fig 2:**
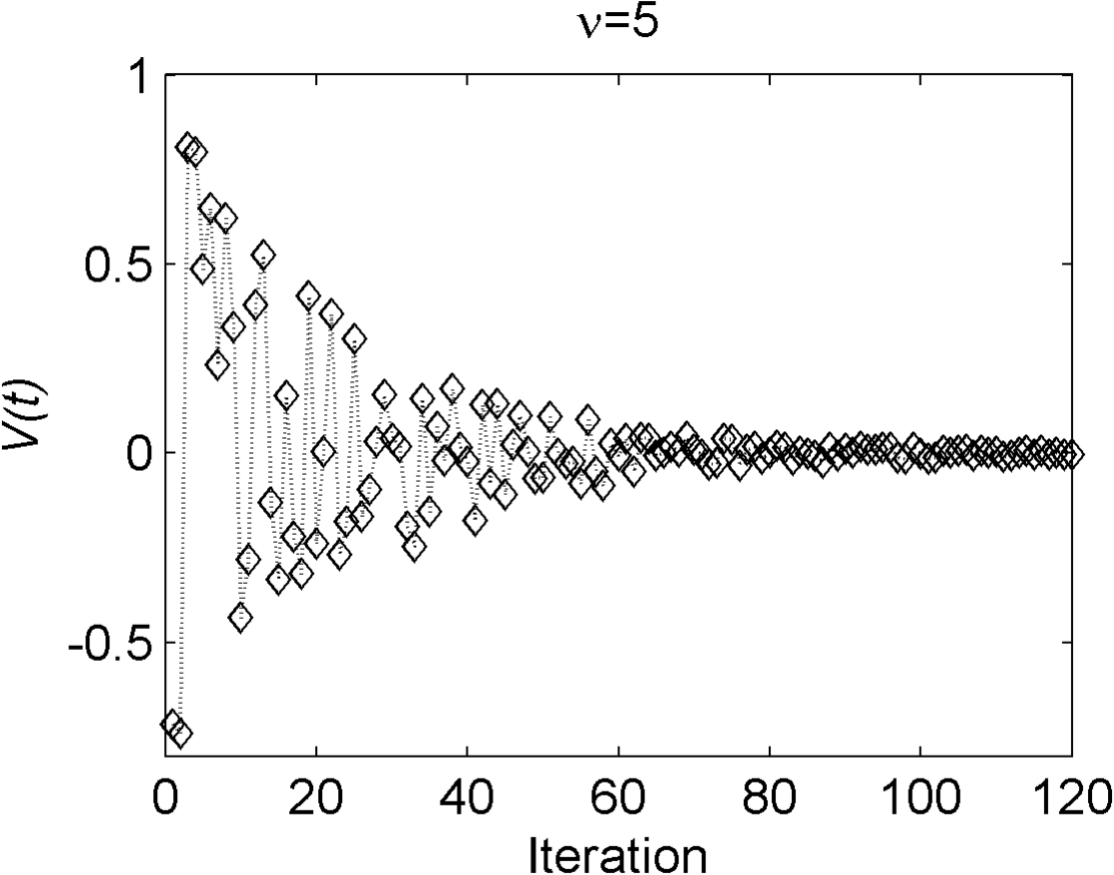
An amplitude of *V*(*t*) along with iteration when *v* = 5.

To overcome such exploitation problem of SSO, this work proposes an exploitation search scheme called VVS rather than a local search. It can refine the quality of the solution by searching the extreme points nearby the global best position. A new variable in a solution after VVS is calculated as Eq (7)

$$VVS(x_{j})=g_{j}+(Ub_{j}-Lb_{j})\times V(t)$$ (7)

where *g*_*j*_ denotes the *j*th variable in *gBest*. *Lb*_*j*_ and *Ub*_*j*_ are the lower and upper bounds of the *j*th variable, respectively. The amplitude constant *V* is a function of time (*t*) as follow:

$$V(t)=\lambda \times \text{exp}(-\nu \frac{iter}{Niter})$$ (8)

where *λ* represents a random number uniformly distributed in [-1,1]. *ν* is a predetermined parameter to handle the amplitude of variables. *Niter* is the total number of iterations and *iter* is the number of current iterations. As shown in Fig 2, with an increase in the number of iterations, *V*(*t*) moves in the plot as vibration wave and decreases toward zero at the last iteration. Hence, the particles search extreme points around the *gBest* position.

The balance between exploration and exploitation is an important criterion that can determine the performance of population-based optimization [24–25]. To tackle this problem, a function of time (*t*), *c*_*r*_(*t*) as Eq (9), is introduced to replace *c*_*r*_ which is a fixed constant in the original UM. This change leads the particles to stochastically explore the search spaces in beginning steps and gradually transform to exploit the extreme points nearby the *gBest* position as the number of iterations increases.

$$c_{r}(t)=c_{r}(0)\times \frac{iter}{Niter}$$ (9)

$$c_{r}(0)=1-C_{g}$$ (10)

However, the modified UM is more complex and consumes more time than the original UM because of the additional VVS. According to the result of preliminary tests as shown in S1 Table, the *pBest* scheme can be discarded in the modified UM to maintain the simplicity and efficiency of the proposed algorithm without affecting the performance of the proposed algorithm. Therefore, the UM of the proposed algorithm is modified as Eq (11)

$$x_{ij}^{t}=\left\{ \begin{array}{l} \;x_{ij}^{t-1}\;if\;\rho \in [0,C_{w})\\ \;g_{j}\;if\;\rho \in [C_{w},C_{g})\\ VVS(x_{j})\;if\;\rho \in [C_{g},C_{g}+c_{r}(t))\\ \;x\;if\;\rho \in [C_{g}+c_{r}(t),1] \end{array}\right.$$ (11)

### Rapid centralized strategy (RCS)

**Fig 3:**
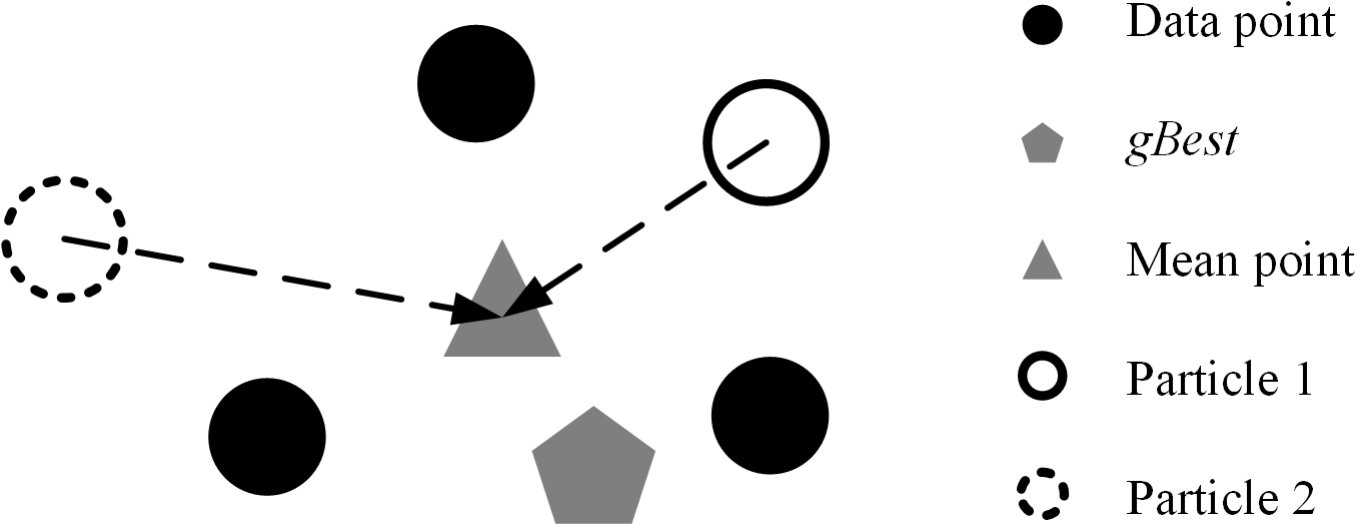
Particles moving behavior in accelerated strategy.

As mentioned in Section 1, the proposed algorithm may take more time to converge. Krishna and Murty [5] employed KMO to determine new cluster centers for all particles in each generation after initial population procedure by using Eq (5). However, overusing the arithmetic average may clamp the particles searching in the solution space because the members in clusters of different particles are the same after running a number of iterations. For example, if two particles in different positions have the same members in clusters, then both of them will move to exactly the same point, which is the mean point of the cluster after conducting KMO, as shown in Fig 3. In this case, particles may keep searching nearby the mean point rather than *gBest*. The computation cost is wasted, and the diversity of population is restricted by arithmetic average.

Some clustering algorithms take advantage of combining with KM and using its result as one of the particles for speeding up the convergence rate [9, 13, 16]. This strategy is named one-from-KM (OFK) in this work. However, these hybrid algorithms may start searching from a local optimum because of the initialization problem of the KM.

In this work, a modified accelerated strategy, RCS, is proposed. RCS is inspired by KMO and can efficiently find a better centroid of a cluster at the initial state and keep the diversity of population. The following two steps constitute RCS:

Step 1: In the initial state, the cluster centroids of all particles are recalculated according to Eq (5), after being generated randomly by the proposed algorithm.
Step 2: In each iteration, only some particles are recalculated by RCS depending on its random number.

Recalculating the centroids of all particles in the initial state can escape from the initialization problem of the KM through the diversity of particles and then obtain a promising initial solution. In step 2, a random number, *rand*, belonging to [0, 1] is generated for each particle after the particle is updated by the modified UM. If *rand* < *β*, then the RCS is implemented to recalculate the cluster centroids of the particle. *β* is a predetermined parameter in the interval [0, 1] to decide the proportion of particles that need to be recalculated the centroids.

**Fig 4:**
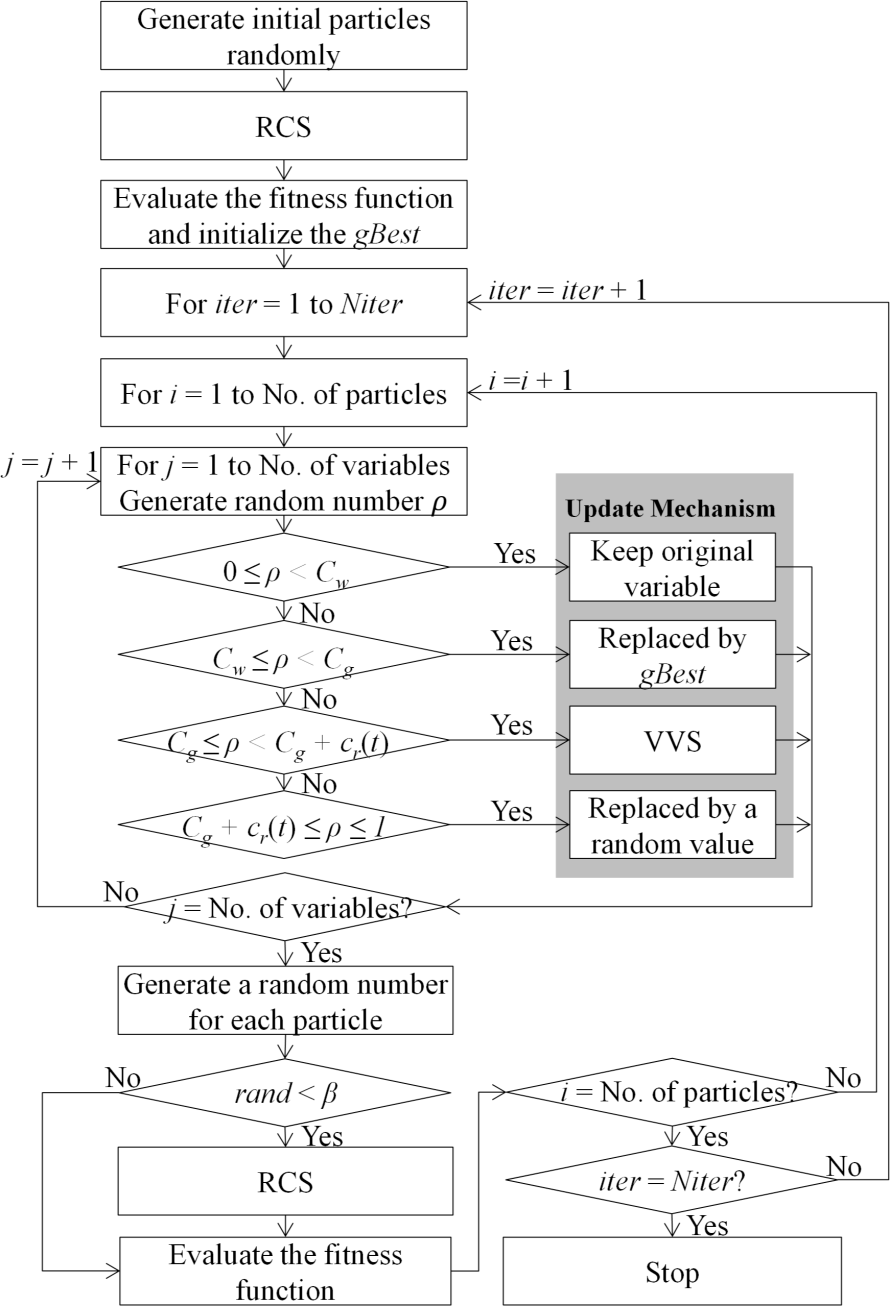
Flowchart of the VSSO-RCS algorithm.

Based on the above description, the proposed algorithm is called VSSO-RCS, and its steps are shown in Fig 4.

## Experiment Results and Discussion

The experiments are composed of two parts. In the first experiment, RCS is compared with other accelerated strategies, including KMO and OFK, to illustrate the effectiveness of the proposed accelerated strategy. In the second experiment, well-known and recently developed population-based algorithms are implemented to evaluate the performance of the proposed algorithm. These population-based algorithms include SSO [17], SSO-ELS [23], BH [3], KSRPSO [13], and GSA-KM [16]. All experiments are executed in MATLAB R2012b on a computer equipped with an Intel 2.4 GHz CPU and 12 GB of memory.

As mentioned in Section 2, this work uses SICD as a criterion to evaluate the performance of all clustering algorithms. SICD is the sum of the distances between objects in the same cluster, as defined in Eq (4). A smaller SICD results in a higher quality solution.

### Data sets

According to the categorization of dataset size described by Kudo and Sklansky [26], the problem can be categorized into three categories in terms of the number of features: small with 0 < *D* ≤ 19, medium with 20 < *D* ≤ 49 and large with *D* ≥ 50. Twelve datasets, which are taken from the UCI repository database [27], cover all categories to test all approaches implemented in this work. The characteristics of these datasets are summarized in Table 1. These datasets have sizes ranging from hundreds to thousands and the feature size ranges from 3 to 60. All datasets contain numeric features with no missing data.

**Table 1:** Characteristics of the considered data sets.

Categorization	Dataset	Features (*D*)	Instances (*N*)	Clusters (*K*)
Small	Vowel	3	871	6
	Iris	4	150	3
	Crude oil	5	56	3
	Cancer	9	683	2
	CMC	9	1473	3
	Glass	9	214	6
	MG Telescope	10	19020	2
	Wine	13	178	3
	EGG eye	15	14980	2
Medium	WDBC	30	569	2
	Ionosphere	34	351	2
Large	Sonar	60	208	2

### Parameter settings

The algorithmic parameters for all approaches are illustrated in Table 2. Parameter settings may influence the quality of results and the settings of each approach are suggested from previous studies. In KSRPSO, the settings of cognition *c*_*1*_ and social *c*_*2*_ parameters are 0.5 and 2.5, respectively. The inertia weight *w* is equal to 0.5×*rand*/2, where *rand* is a uniformly generated random number between 0 and 1. Three parameters, *f*, *d* and *a*, are set to 0.2, 0.2 and 0.8 respectively, controlling the selective particle regeneration mechanism for local search [13]. The parameters in SSO, SSO-ELS and SSO-RCS, *C*_*w*_, *C*_*p*_ and *C*_*g*_, are all set at 0.1, 0.4 and 0.9, respectively [17, 23]. In VSSO-RCS, *C*_*w*_, *C*_*g*_, *ν* and *β* are set at 0.2, 0.9, 10 and 0.1, respectively. Based on the preliminary test this parameter setting of VSSO-RCS provides a good chance of finding the global optimal solution. Two parameters in GSA-KM, the initial gravitational constant *G*_*0*_ and *α*, are set at 100 and 20, respectively [16, 28]. BH has no parameters need to be set [3].

**Table 2:** Value of parameters in six algorithms.

Parameter^a^	KSRPSO	SSO-based	VSSO-RCS	GSA-KM
	Value			
*c_1_, c_2_*	0.5, 2.5	-	-	-
*w*	0.5×*rand*/2	-	-	-
f, *d, a*	0.2, 0.2, 0.8	-	-	-
*C_w_, C_p_, C_g_*	-	0.1, 0.4, 0.9	0.2, -, 0.9	-
*v, β*	-	-	10, 0.1	-
*G_0_, α*	-	-	-	100, 20

^a^ There is no parameter need to be set in BH [3].

For each run, the number of iterations and population size of each approach depends on the number of features and clusters of each dataset. 10×*Ψ* iterations and 3×*Ψ* population size are performed, and *Ψ* is equal to *D*×*K*. [11, 12].

### Results and discussion

#### Experiment 1: Evaluation of RCS

KM, SSO, SSO-KMO, SSO-OFK and SSO-RCS are implemented to demonstrate the effectiveness of the proposed RCS. SSO-KMO, SSO-OFK, and SSO-RCS represent SSO that adopted the accelerated strategies KMO, OFK, and RCS, respectively. Four datasets are selected among three categories of datasets for this experiment, including Cancer, Glass, Ionosphere, and Sonar. The experimental results summarized in Tabel 3 include SICD and CPU time (CT). SICD is given in terms of the best, average, worst, and standard deviation (Std.) of the obtained solutions after 30 runs. CPU time is a record of the average proceeding time of 30 runs.

**Table 3:** Results of three accelerated strategies.

Dataset	Criteria	KM	SSO	SSO-KMO	SSO-OFK	SSO-RCS
Cancer	Best	2,986.96	2,965.13	2,965.56	2,964.82	2,964.93
	Avg.	2,987.84	2,967.87	2,967.11	2,966.65	**2,965.91**
	Worst	2,988.43	2,975.96	2,969.20	2,970.40	2,967.93
	Std.	0.73	2.55	1.05	1.40	0.82
	CT(s)	1.45	5.52	10.84	5.79	5.83
Glass	Best	213.24	213.52	211.53	211.70	211.34
	Avg.	223.58	223.85	220.55	214.71	**212.11**
	Worst	253.83	240.66	243.43	218.57	215.60
	Std.	10.21	9.74	10.00	1.65	1.01
	CT(s)	2.75	45.64	85.25	48.05	48.42
INSP	Best	796.33	811.75	795.90	795.42	795.13
	Avg.	796.40	817.62	796.23	796.10	**795.37**
	Worst	796.47	824.36	796.66	796.33	795.59
	Std.	0.07	3.72	0.28	0.28	0.15
	CT(s)	1.88	110.06	197.01	108.65	114.02
Sonar	Best	234.77	235.25	240.00	234.72	234.51
	Avg.	235.10	238.32	240.42	235.06	**234.58**
	Worst	235.21	240.32	241.66	235.21	234.60
	Std.	0.15	2.33	0.50	0.20	0.03
	CT(s)	2.29	664.94	693.93	432.56	425.46

As shown in Table 3, where the best average values are shown in bold, SSO without any accelerated strategy produces the worst solution on all selected datasets in comparison with SSO-KMO, SSO-OFK, and SSO-RCS. This result confirms that the three accelerated strategies are functional and facilitate SSO to find better solutions. SSO-KMO consumes more time and obtains worse solution than SSO-RCS. This result proves that RCS modified from KMO not only reduces the computational cost but also enhances the performance of SSO. As expected, SSO-OFK is faster than the other strategies. However, the solutions obtained by SSO-OFK are all worse than those obtained by SSO-RCS. The results reveal that SSO-RCS yields higher quality solutions on all selected datasets than SSO-KMO and SSO-OFK. Obviously, RCS outperforms other strategies in this experiment.

**Fig 5:**
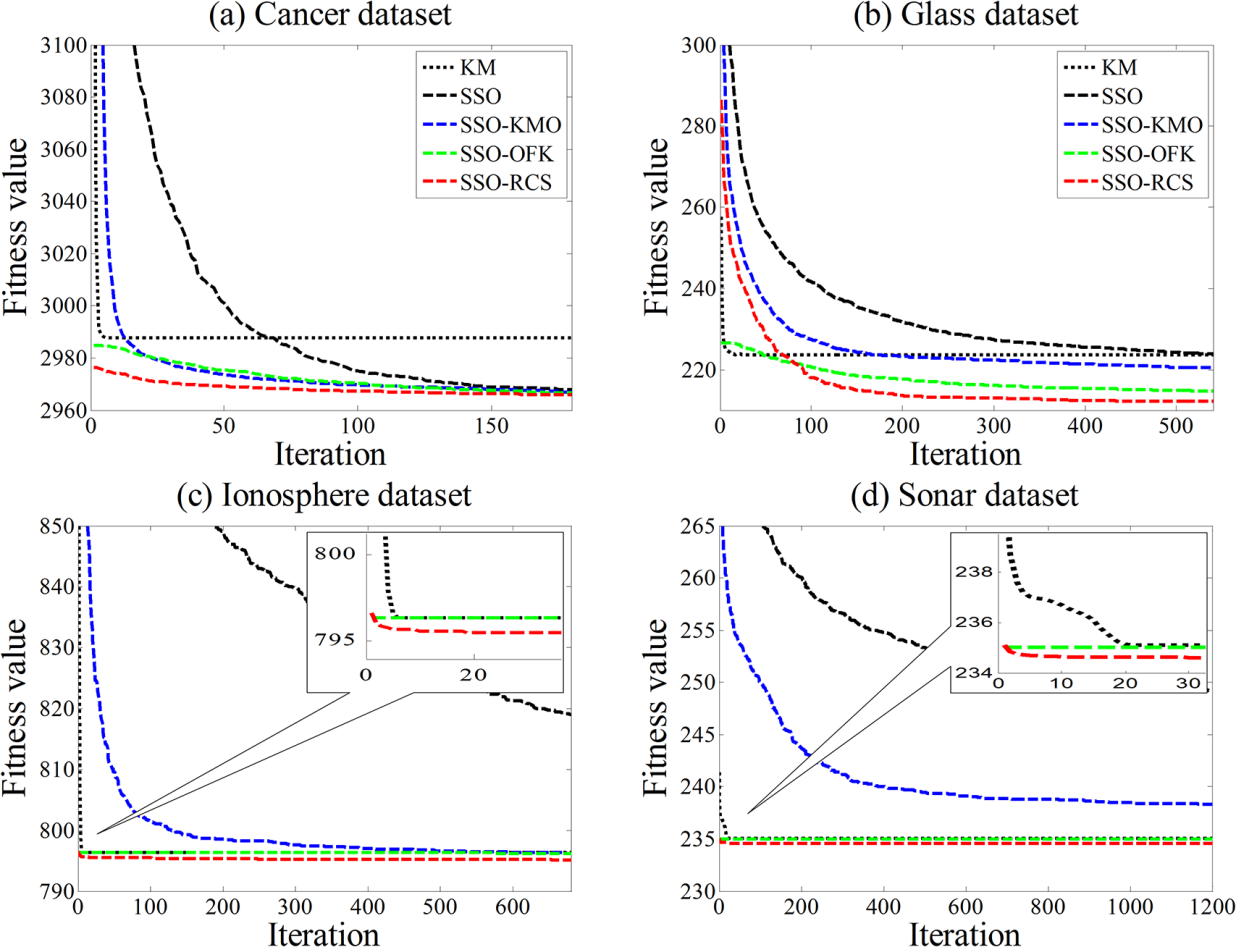
Convergence comparison of different accelerated strategies.

Fig 5 depicts the progress of the average *gBest* over 30 runs, providing insights into the convergence behavior of SSO-KMO, SSO-OFK, and SSO-RCS. For all datasets, SSO exhibits the worst convergence pattern. SSO-KMO cannot obtain any benefit of yielding initial solutions. Furthermore, it displays a fast but premature convergence to a local optimum on Glass, Ionosphere and Sonar dataset, as shown in Fig 5(B), 5(C) and 5(D). This result is understandable because the global search capability depends on the diversity of population. KMO recalculates the centroids of all particles at each iteration using the arithmetic average. After a number of iterations, the overuse of the arithmetic average may backfire and compel the particle to be the same as its predecessor or neighbors. As a result, KMO may be disabled, and the population diversity would be diminished.

OFK using the result of KM as one of the initial particles can offer SSO-OFK promising initial solutions that lead to initially converging faster than SSO-KMO. However, the initial solutions obtained from KM may suffer from local optimum. Therefore, *gBest* and *pBest* are initialized from a pitfall and seriously affect the capability of global search, as shown in Fig 5(B), 5(C) and 5(D).

SSO-RCS demonstrates a consistent performance pattern among all the considered accelerated strategies. RCS not only produces promising initial solutions but also aids SSO to find higher quality solutions than SSO-KMO and SSO-OFK on all of the selected datasets. Results show that the proposed RCS is an ideal choice for accelerating convergence speed in clustering algorithms.

#### Experiment 2: Evaluation of VSSO-RCS

Well-known and recent population-based algorithms including SSO, SSO-RCS, SSO-ELS, BH, KSRPSO, and GSA-KM are implemented to demonstrate the effectiveness of the proposed algorithm, VSSO-RCS. Experimental results tested on 12 benchmark datasets are summarized in Tables 4–6, where the best values are shown in bold and all results are obtained after 30 runs.

**Table 4:** Results obtained by the algorithms on Vowel, Iris, Crude oil and Cancer datasets.

Dataset	Criteria	SSO	SSO-RCS	SSO-ELS	BH	KSRPSO	GSA-KM	VSSO-RCS
Vowel	Best	149,247.64	149,021.16	149,096.69	**148,967.24**	149,089.96	149,076.71	**148,967.24**
	Avg	150,421.69	149,315.13	150,118.24	151,684.62	151,758.39	152,289.92	**149,148.08**
	Worst	154,119.51	150,267.43	153,232.13	168,379.82	170,433.64	158,612.03	**150,139.66**
	Std	1,249.07	394.08	863.56	5,100.30	4,205.04	2,947.95	**336.16**
	CT	6.54	6.97	10.86	**6.13**	6.65	13.38	6.44
Iris	Best	96.75	96.67	96.72	**96.66**	96.68	**96.66**	**96.66**
	Avg	97.11	96.73	97.17	**96.66**	98.98	96.71	**96.66**
	Worst	98.06	96.82	97.94	96.71	127.67	97.22	**96.66**
	Std	0.35	0.04	0.42	0.01	6.96	0.13	**0.00**
	CT	0.70	0.74	1.20	**0.61**	0.75	1.85	**0.61**
Crude oil	Best	277.25	277.25	277.24	**277.21**	277.22	277.21	**277.21**
	Avg	277.53	277.35	277.99	277.27	277.35	277.63	**277.26**
	Worst	278.21	277.42	293.58	**277.30**	277.86	285.76	277.36
	Std	0.25	**0.04**	2.95	**0.04**	0.13	1.56	0.05
	CT	0.99	1.06	1.64	**0.83**	1.02	3.76	**0.83**
Cancer	Best	2,965.13	2,964.93	2,964.95	2,964.39	2,964.86	2,965.00	**2,964.39**
	Avg	2,967.87	2,965.91	2,967.05	2,964.40	2,966.32	2,973.42	**2,964.39**
	Worst	2,975.96	2,967.93	2,970.83	2,964.41	2,969.62	2,985.84	**2,964.39**
	Std	2.55	0.82	1.32	0.00	1.24	6.57	**0.00**
	CT	5.52	5.83	10.03	**5.16**	5.90	11.28	5.39

**Table 5:** Results obtained by the algorithms on CMC, Glass, MG Telescope and Wine datasets.

Dataset	Criteria	SSO	SSO-RCS	SSO-ELS	BH	KSRPSO	GSA-KM	VSSO-RCS
CMC	Best	5,533.46	5,532.77	5,532.76	5,532.19	5,532.36	5,532.19	**5,532.18**
	Avg	5,535.93	5,533.45	5,534.84	5,532.24	5,532.87	5,532.19	**5,532.18**
	Worst	5,540.84	5,534.59	5,539.57	5,532.45	5,533.59	5,532.19	**5,532.18**
	Std	1.77	0.45	1.48	0.05	0.32	**0.00**	**0.00**
	CT	27.90	28.66	46.22	**26.10**	27.45	54.62	27.12
Glass	Best	213.52	211.34	211.19	237.65	**210.36**	213.11	210.43
	Avg	223.85	212.11	218.38	260.93	217.87	218.52	**211.31**
	Worst	240.66	215.60	235.35	269.64	245.32	227.58	**214.81**
	Std	9.74	**1.01**	8.50	8.36	7.91	3.60	1.78
	CT	45.64	48.42	78.82	38.66	**36.94**	508.16	39.26
MG Telescope	Best	1,623,698.99	1,623,296.10	1,623,772.73	1,623,042.28	1,623,322.11	**1,623,042.27**	1,623,042.28
	Avg	1,631,490.90	1,625,505.00	1,629,648.90	1,623,042.31	1,627,770.46	**1,623,042.27**	1,623,045.45
	Worst	1,637,591.98	1,632,364.00	1,635,628.40	1,623,042.38	1,635,781.99	**1,62,3042.27**	1,623,072.86
	Std	4,685.46	3,544.38	3,877.04	0.03	4,191.48	**0.00**	9.63
	CT	1145.16	1175.22	1709.08	**988.84**	1211.86	1341.66	1084.09
Wine	Best	16,292.44	16,292.45	16,292.43	16,292.19	16,292.22	16,292.67	**16,292.18**
	Avg	16,294.07	16,293.86	16,294.14	**16,292.71**	16,292.78	16,293.90	16,292.76
	Worst	16,296.90	16,295.72	16,295.67	**16,294.17**	16,294.47	**16,294.17**	**16,294.17**
	Std	1.09	0.89	1.05	0.70	0.67	**0.45**	0.82
	CT	20.00	21.65	34.01	**17.64**	18.33	141.91	18.08

**Table 6:** Results obtained by the algorithms on EGG eye, WDBC, INSP and Sonar datasets.

Dataset	Criteria	SSO	SSO-RCS	SSO-ELS	BH	KSRPSO	GSA-KM	VSSO-RCS
EGG eye	Best	8,032,669.11	5,644,264.31	2,385,500.94	**2,354,713.85**	3,010,467.48	2,778,514.06	2,354,756.19
	Avg	16,361,259.24	14,464,277.41	2,505,596.68	2,586,299.09	3,210,719.24	2,791,675.62	**2,354,849.12**
	Worst	26,910,999.74	29,333,498.64	2,762,809.53	3,214,000.36	3,456,696.67	2,867,748.16	**2,355,129.21**
	Std	6,668,788.32	6,757,831.04	108,461.85	271,990.85	114,894.72	27,242.75	**129.73**
	CT	856.77	874.94	1116.37	850.98	880.40	912.04	**849.99**
WDBC	Best	149,474.46	149,474.20	149,474.40	**149,473.86**	149,473.89	**149,473.86**	**149,473.86**
	Avg	149,480.77	149,477.49	149,479.23	**149,473.86**	149,474.13	**149,473.86**	**149,473.86**
	Worst	149,494.82	149,483.18	149,493.45	149,473.87	149,474.62	**149,473.86**	**149,473.86**
	Std	5.93	2.76	4.10	**0.00**	0.20	**0.00**	**0.00**
	CT	94.57	105.77	185.15	90.91	**90.58**	732.59	90.76
INSP	Best	814.52	794.96	794.32	793.92	793.78	**793.71**	**793.71**
	Avg	819.07	795.13	794.86	794.30	793.87	**793.71**	**793.71**
	Worst	827.72	795.30	796.37	795.34	794.02	**793.71**	793.72
	Std	4.76	0.10	0.61	0.42	0.07	**0.00**	**0.00**
	CT	105.74	114.02	195.46	105.38	**95.97**	1,171.57	96.35
Sonar	Best	247.73	234.51	238.85	234.22	233.77	**233.76**	**233.76**
	Avg	249.19	234.58	239.87	245.02	233.86	**233.76**	**233.76**
	Worst	251.12	234.60	245.27	266.59	234.08	**233.76**	233.77
	Std	1.11	0.03	1.93	14.97	0.09	**0.00**	**0.00**
	CT	395.85	425.46	650.07	347.83	**301.61**	11,132.94	328.31

As illustrated in Tables 4–6, VSSO-RCS produces better results than SSO-RCS on all of the datasets. This result proves that the proposed exploitation scheme VVS plays its prescribed roles in exploitation search and can refine the solutions. For the Vowel, Crude oil, Cancer, CMC, Glass and EGG eye datasets, the average values obtained by VSSO-RCS are 149148.08, 277.26, 2964.39, 5532.18, 211.31 and 2354849.12, respectively, which are better than those obtained by the other algorithms. For the Cancer, CMC, EGG eye and WDBC datasets, the worst values obtained by VSSO-RCS are 2964.39, 5532.18, 2355129.21 and 149473.86, respectively, which are better than the best values yielded by the other test algorithms except for BH and GSA-KM. This result means that other SSO-based algorithms and KSRPSO are powerless to achieve those values even once within 30 runs. Moreover, the standard deviation on the Iris, Cancer, CMC, WDBC, Ionosphere, and Sonar datasets are 0.00, which means that the proposed algorithm is more reliable than the other algorithms.

In terms of CPU time, the proposed algorithm is faster than other SSO-based algorithms. This advantage is attributed to the modified UM of VSSO-RCS by discarding the *pBest* scheme. GSA-KM is time consuming because of its computational complexity. In general, BH and VSSO-RCS take less processing time than KSRPSO on small and medium size datasets. KSRPSO has superiority on large size dataset.

#### The statistical analysis

A nonparametric statistical analysis, the Friedman test, is conducted to confirm whether the proposed VSSO-RCS offers a significant improvement. If statistically significant differences exist among all algorithms, then the Holm’s method is employed as a post hoc test to compare the proposed algorithm (control algorithm) and the other algorithms. The significance level is set to *α* = 0.05 to determine whether or not the hypothesis is rejected in all cases. These tests are detailed in [29].

Table 7 reports the average ranks computed through the Friedman test based on the average values of SICD. The table shows that the average rank of the proposed VSSO-RCS is the smallest among the algorithms. Therefore, the proposed method is the best performing one, followed by BH, SSO-RCS, GSA-KM, KSRPSO, SSO-ELS, and SSO, successively.

**Table 7:** Results of Friedman ranks on the average of SICD.

Dataset	SSO	SSO-RCS	SSO-ELS	BH	KSRPSO	GSA-KM	VSSO-RCS
Vowel	4.00	2.00	3.00	5.00	6.00	7.00	1.00
Iris	5.00	4.00	6.00	1.50	7.00	3.00	1.50
Crude oil	5.00	3.50	7.00	2.00	3.50	6.00	1.00
Cancer	6.00	3.00	5.00	2.00	4.00	7.00	1.00
CMC	7.00	5.00	6.00	3.00	4.00	2.00	1.00
Glass	6.00	2.00	4.00	7.00	3.00	5.00	1.00
MG T	7.00	4.00	6.00	2.00	5.00	1.00	3.00
Wine	6.00	4.00	7.00	1.00	3.00	5.00	2.00
EGG eye	7.00	6.00	2.00	3.00	5.00	4.00	1.00
WDBC	7.00	5.00	6.00	2.00	4.00	2.00	2.00
Ionosphere	7.00	6.00	5.00	4.00	3.00	1.50	1.50
Sonar	7.00	4.00	5.00	6.00	3.00	1.50	1.50
Average	6.17	4.04	5.17	3.21	4.21	3.75	1.46

The *p*-Values computed through the Friedman test is given in Table 8, suggesting significant differences in the average values of SICD among the considered approaches.

**Table 8:** Results of Friedman tests on the average of SICD.

Method	Statistical value	*p*-Value	Hypothesis
Friedman	34.07	0.000	Rejected

To determine sufficient statistical differences between VSSO-RCS and the remaining algorithms, the Holm’s method is conducted as a post hoc test.

Table 9 shows that all *p*-values are smaller than α = 0.05, which indicates that the control algorithm VSSO-RCS is statistically better than SSO, SSO-RCS, SSO-ELS, BH, KSRPSO, and GSA-KM in term of SICD.

**Table 9:** Results of the post hoc test on the average of SICD.

Method	*z*-Value	*p*-Value	Hypothesis
SSO	5.339	0.000	Rejected
VSSO-RCS	2.929	0.003	Rejected
SSO-ELS	4.205	0.000	Rejected
BH	1.984	0.047	Rejected
KSRPSO	3.118	0.002	Rejected
GSA-KM	2.598	0.009	Rejected

The same procedure is conducted to check whether or not significant differences in CPU time exist between the clustering algorithms. The results are shown in Tables 10–12. The Friedman’s test reveals that the proposed algorithm is ranked second behind BH and that statistically significant differences in the average CPU time exist among the algorithms.

Table 12 indicates that VSSO-RCS is more efficient than SSO-RCS, SSO-ELS, and GSA-KM. No significant difference is found among SSO, KSRPSO, BH, and VSSO-RCS.

**Table 10:** Results of Friedman ranks on the average of CPU time.

Dataset	SSO	SSO-RCS	SSO-ELS	BH	KSRPSO	GSA-KM	VSSO-RCS
Vowel	3.00	5.00	6.00	1.00	4.00	7.00	2.00
Iris	3.00	4.00	6.00	1.50	5.00	7.00	1.50
Crude oil	3.00	5.00	6.00	1.50	4.00	7.00	1.50
Cancer	3.00	4.00	6.00	1.00	5.00	7.00	2.00
CMC	4.00	5.00	6.00	1.00	3.00	7.00	2.00
Glass	4.00	5.00	6.00	2.00	1.00	7.00	3.00
MG T	3.00	4.00	7.00	1.00	5.00	6.00	2.00
Wine	4.00	5.00	6.00	1.00	3.00	7.00	2.00
EGG eye	3.00	4.00	7.00	2.00	5.00	6.00	1.00
WDBC	4.00	5.00	6.00	3.00	1.00	7.00	2.00
INSP	4.00	5.00	6.00	3.00	1.00	7.00	2.00
Sonar	4.00	5.00	6.00	3.00	1.00	7.00	2.00
Average	3.50	4.67	6.17	1.75	3.17	6.83	1.92

**Table 11:** Results of Friedman tests on the average of CPU time.

Method	Statistical value	*p*-Value	Hypothesis
Friedman	60.464	0.000	Rejected

**Table 12:** Results of the post hoc test on the average of SICD.

Method	*z*-Value	*p*-Value	Hypothesis
SSO	1.795	0.073	Not rejected
VSSO-RCS	3.118	0.002	Rejected
SSO-ELS	4.819	0.000	Rejected
BH	0.189	1.150	Not rejected
KSRPSO	1.417	0.156	Not rejected
GSA-KM	5.575	0.000	Rejected

The results of empirical and statistical analyses show that VSSO-RCS is not that superior in CPU time compared with SSO, BH and KSRPSO. However, VSSO-RCS exhibits promising and effective clustering performance.

#### Empirical analysis of algorithm efficiency

The results from the previous subsection demonstrate that the proposed algorithm can perform better than its competitors in terms of the quality of solutions. Also, it can be observed that the problem size may impact the computation time. To better observe the effect of the number of data instances and features on the proposed method, two groups of artificial datasets are generated. As shown in Table 13, the first group varies the number of instances from 16000 to 2048000 at the fixed number of features (i.e. *D* = 6) for evaluating the effect of instance size. The second group varies the number of features from 400 to 51200 at the fixed number of instances (*N* = 1000) for observing the effect of feature size as shown in Table 14. The number of clusters in both of the two groups is fixed to *K* = 2. The proposed method performs 10 independent runs on each dataset with 100 iterations and 30 population size.

**Table 13:** Empirical analysis result of varying in number of data instances.

*i*	1	2	3	4	5	6	7	8
*N* (*D* = 6)	16000	32000	64000	128000	256000	512000	1024000	2048000
Max CT	15.21	29.72	56.83	121.20	246.08	496.77	999.67	2149.43
Mean CT	14.47	28.49	55.80	119.06	241.86	484.74	966.41	1958.85
Min CT	13.85	27.38	54.90	116.95	237.07	473.70	945.32	1989.48
Std CT	0.48	0.73	0.69	1.26	2.77	6.65	18.57	51.56
Ratio^a^		1.97	1.96	2.13	2.03	2.00	1.99	2.03

^a^Ratio = Mean CT_*i*_ / Mean CT_i-1_

**Table 14:** Empirical analysis result of varying in number of data features.

*i*	1	2	3	4	5	6	7	8
*D* (*N* = 1000)	400	800	1600	3200	6400	12800	25600	51200
Max CT	55.82	109.48	217.14	434.14	881.76	1764.43	3592.34	7413.43
Mean CT	54.76	108.70	214.54	431.66	877.09	1754.31	3574.15	7227.01
Min CT	53.43	107.58	211.54	428.05	871.33	1727.27	3557.24	7230.68
Std CT	0.71	0.60	1.95	2.07	2.88	10.68	10.60	54.29
Ratio		1.99	1.97	2.01	2.03	2.00	2.04	2.02

**Fig 6:**
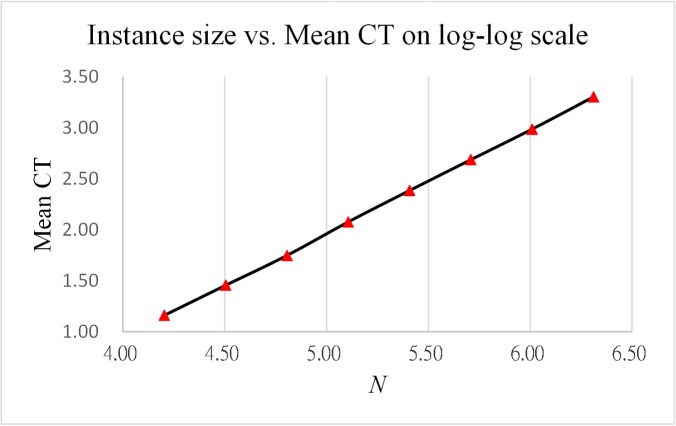
Log-log plot of CPU time vs. instance size.

**Fig 7:**
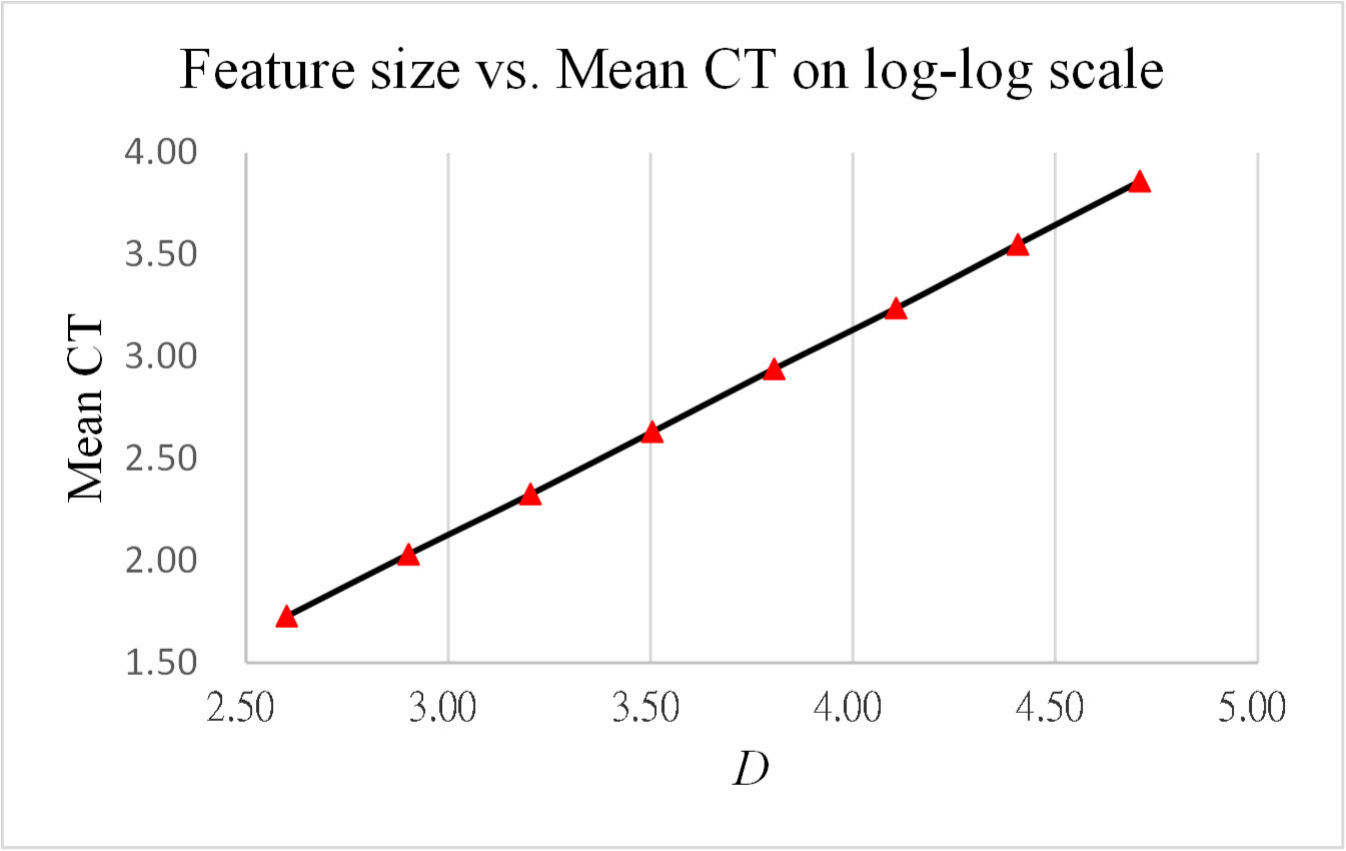
Log-log plot of CPU time vs. feature size.

As reported in Tables 13 and 14, the results obtained from the two analysis cases indicate that the ratios of CPU time increase both converge to 2. Fig 6 and Fig 7 show log-log plots of CPU time vs. instance size and vs. feature size, respectively, both indicating that the computation time grows with the size of instance and feature in a linear and stable manner.

## Conclusions

The proposed VSSO-RCS is a modified version of simplified swarm optimization to effectively and efficiently tackle the clustering problems. VVS overcomes the exploitation problem in SSO and facilitates VSSO-RCS to refine the quality of solution. VSSO-RCS also converges fast to an optimum solution by adopting RCS.

To assess the performance of VSSO-RCS, two experiments are conducted. First, RCS is compared with other two powerful accelerated strategies. RCS not only can obtain promising initial solutions but also converge more efficiently than KMO and OFK. Second, VSSO-RCS is compared with state-of-the-art population-based algorithms on 12 benchmark datasets. Results reveal that VSSO-RCS is superior to its competitors from the perspective of solution quality and is efficient in terms of the processing time required.

Our results on empirical analysis suggest that instance and feature size both affect the computation efficiency of the proposed algorithm. As the problem size grows, it adds more challenges to the computation time of our proposed method, especially for dealing with very large-size problems (i.e. big data) [30]. Due to such aspect, our future research will focus on the following:

1. Apply effective techniques [31–35] which can reduce the search space into the proposed algorithm to mitigate such an issue.
2. Modify the proposed method in a parallel or distributed form [36–38] to improve the computation efficiency.

In addition, it is also worth exploring other potential application, such as classification, fully utilize the VSSO-RCS.

## Supporting Information

S1 Table Results of preliminary tests. (DOCX)
