# Supplementary material for: Accelerated Simplified Swarm Optimization with Exploitation Search Scheme for Data Clustering
Source: PLoS One. 2015 Sep 8;10(9):e0137246. doi: 10.1371/journal.pone.0137246 (PMC4562660; doi:10.1371/journal.pone.0137246)
Supplement: S1 Table — (DOCX) [file pone.0137246.s001.docx]

| Dataset | Criteria | ^a^VSSO-RCSp | VSSO-RCS | Dataset | VSSO-RCSp | VSSO-RCS | Dataset | VSSO-RCSp | VSSO-RCS |
| --- | --- | --- | --- | --- | --- | --- | --- | --- | --- |
| Vowel | Best | **148,967.24** | **148,967.24** | CMC | **5,532.18** | **5,532.18** | EGG | 2,354,781.15 | **2,354,756.19** |
|  | Avg | 149,180.91 | **149,148.08** |  | 5,532.19 | **5,532.18** |  | 2,354,966.32 | **2,354,849.12** |
|  | Worst | 150,139.80 | **150,139.66** |  | 5,532.20 | **5,532.18** |  | 2,355,203.85 | **2,355,129.21** |
|  | Std | 340.25 | **336.16** |  | 0.00 | 0.00 |  | 137.52 | **129.73** |
|  | CT | 7.47 | **6.44** |  | 28.12 | **27.12** |  | 912.55 | **849.99** |
| Iris | Best | **96.66** | **96.66** | Glass | **210.01** | 210.43 | WDBC | **149,473.86** | **149,473.86** |
|  | Avg | **96.66** | **96.66** |  | **210.71** | 211.31 |  | **149,473.86** | **149,473.86** |
|  | Worst | **96.66** | **96.66** |  | 214.83 | **214.81** |  | **149,473.86** | **149,473.86** |
|  | Std | **0.00** | **0.00** |  | **1.12** | 1.78 |  | **0.00** | **0.00** |
|  | CT | 0.68 | **0.61** |  | 42.84 | **39.26** |  | 94.17 | **90.76** |
| Crude oil | Best | **277.21** | 277.21 | MG T | 1,623,042.36 | **1,623,042.28** | INSP | 793.72 | **793.71** |
|  | Avg | **277.24** | 277.26 |  | 1,623,324.91 | **1,623,045.45** |  | 793.73 | **793.71** |
|  | Worst | **277.30** | 277.36 |  | 1,624,213.63 | **1,623,072.86** |  | 793.74 | **793.72** |
|  | Std | **0.04** | 0.05 |  | 411.24 | **9.63** |  | 0.01 | **0.00** |
|  | CT | 0.95 | **0.83** |  | 1182.72 | **1084.09** |  | 103.14 | **96.35** |
| Cancer | Best | **2,964.39** | **2,964.39** | Wine | **16,292.18** | **16,292.18** | Sonar | 233.77 | **233.76** |
|  | Avg | **2,964.39** | **2,964.39** |  | 16,292.95 | **16,292.76** |  | 233.78 | **233.76** |
|  | Worst | **2,964.39** | **2,964.39** |  | **16,294.17** | **16,294.17** |  | 233.78 | **233.77** |
|  | Std | **0.00** | **0.00** |  | **0.82** | **0.82** |  | 0.00 | **0.00** |
|  | CT | 5.81 | **5.39** |  | 19.07 | **18.08** |  | 379.04 | **328.31** |

^a^VSSO-RCSp is the clustering algorithm that VSSO-RCS without discarding *pBest*.
